# NPC1L1 Facilitates Sphingomyelin Absorption and Regulates Diet-Induced Production of VLDL/LDL-associated S1P

**DOI:** 10.3390/nu12092641

**Published:** 2020-08-30

**Authors:** Yoshihide Yamanashi, Tappei Takada, Hideaki Yamamoto, Hiroshi Suzuki

**Affiliations:** Department of Pharmacy, The University of Tokyo Hospital, Faculty of Medicine, The University of Tokyo, Tokyo 113-8655, Japan; yoshihide-tky@umin.ac.jp (Y.Y.); yamahide1013@gmail.com (H.Y.); suzukihi-tky@umin.ac.jp (H.S.)

**Keywords:** absorption, apoM, Niemann-Pick C1-Like 1, sphingomyelin, sphingosine-1-phosphate

## Abstract

Niemann-Pick C1-Like 1 (NPC1L1) is a cholesterol importer and target of ezetimibe, a cholesterol absorption inhibitor used clinically for dyslipidemia. Recent studies demonstrated that NPC1L1 regulates the intestinal absorption of several fat-soluble nutrients, in addition to cholesterol. The study was conducted to reveal new physiological roles of NPC1L1 by identifying novel dietary substrate(s). Very low-density lipoprotein and low-density lipoprotein (VLDL/LDL) are increased in Western diet (WD)-fed mice in an NPC1L1-dependent manner, so we comprehensively analyzed the NPC1L1-dependent VLDL/LDL components. Apolipoprotein M (apoM), a binding protein of sphingosine-1-phosphate (S1P: a lipid mediator), and S1P were NPC1L1-dependently increased in VLDL/LDL by WD feeding. S1P is metabolized from sphingomyelin (SM) and SM is abundant in WD, so we focused on intestinal SM absorption. In vivo studies with Npc1l1 knockout mice and in vitro studies with NPC1L1-overexpressing cells revealed that SM is a physiological substrate of NPC1L1. These results suggest a scenario in which dietary SM is absorbed by NPC1L1 in the intestine, followed by SM conversion to S1P and, after several steps, S1P is exported into the blood as the apoM-bound form in VLDL/LDL. Our findings provide insight into the functions of NPC1L1 for a better understanding of sphingolipids and S1P homeostasis.

## 1. Introduction

Sphingolipids constitute a class of lipids that are essential components of membranes in eukaryotes. In addition to their primary roles in membrane formation, some bioactive sphingolipids and their metabolites (e.g., sphingomyelin (SM) and sphingosine-1-phosphate (S1P)) have attracted attention as important regulators of multiple cellular behaviors and responses, including cell proliferation, cell death, and immune responses [1]. Recently, numerous studies have demonstrated that changes in the dietary intake of sphingolipids influence the progression and/or onset of several lifestyle-related diseases, cancer, and inflammation, indicating that dietary sphingolipids are physiologically important [2,3]. However, the detailed molecular mechanisms of the intestinal absorption of sphingolipids remain unclear.

Niemann-Pick C1-Like 1 (NPC1L1) is a transmembrane protein highly expressed in the intestine, particularly in the luminal membrane of intestinal epithelial cells, in mammals, including in mice and humans [4]. NPC1L1 functions as a cholesterol importer to mediate intestinal cholesterol absorption [4,5], and is reported to be a target molecule of ezetimibe, a cholesterol absorption inhibitor used clinically for dyslipidemia [6]. Our recent studies demonstrated that NPC1L1 is involved in intestinal absorption of fat-soluble vitamins, such as vitamin E and vitamin K_1_, in addition to cholesterol [7,8,9]. Given the large difference in the chemical structures of these vitamins and cholesterol, NPC1L1 may have relatively broad substrate specificity and regulate the intestinal absorption of various dietary lipids.

In this study, based on previous observations that very low-density lipoprotein (VLDL) and low-density lipoprotein (LDL) increase after Western diet (WD) feeding in Npc1l1 wild-type (Npc1l1^WT^) mice but not in Npc1l1 knockout (Npc1l1^KO^) mice [10], we analyzed the NPC1L1-dependent VLDL/LDL components and found that NPC1L1 regulates VLDL/LDL-associated S1P levels. Moreover, we also revealed that intestinal absorption of SM, a precursor of S1P, is controlled by the NPC1L1-mediated pathway. These results suggest a scenario in which dietary SM is absorbed by NPC1L1 in the intestine, followed by SM conversion to S1P which, after several steps, is exported into the blood in VLDL/LDL-associated forms.

## 2. Materials & Methods

### 2.1. Animals

**Figure 1:**
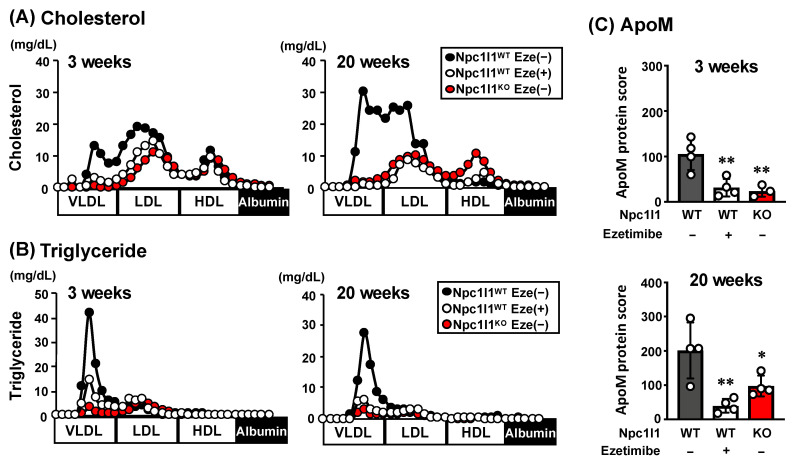
Characterization of Niemann-Pick C1-Like 1 (NPC1L1)-dependent very low-density lipoprotein and low-density lipoprotein (VLDL/LDL) particles in Npc1l1^WT^ or Npc1l1^KO^ mice. (**A**) Cholesterol and (**B**) triglyceride concentrations in lipoprotein fractions, and (**C**) protein score, which reflects protein abundance and degree of confidence in protein identification, of apoM in VLDL/LDL fractions, collected from Npc1l1^WT^ or Npc1l1^KO^ male mice (initially 8–10 weeks old) fed a Western diet (WD) for the indicated periods in the absence (−) or presence (+) of ezetimibe (Eze). Representative lipoprotein profiles from two independent experiments with two mice in each group are shown. Bar graphs represent the mean ± S.D. Each dot on the bar graphs represents data from a single mouse (n = 3–4). * *p* < 0.05; ** *p* < 0.01, significant difference by Dunnett’s test compared with Npc1l1^WT^ Eze (−) mice.

**Figure 2:**
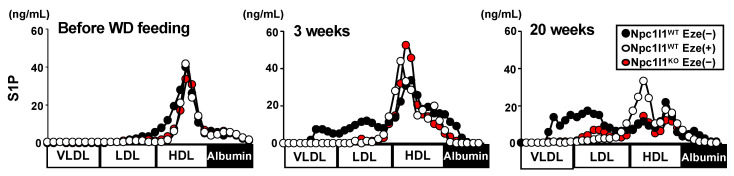
Sphingosine-1-phosphate (S1P) distribution in lipoprotein fractions. Sphingosine-1-phosphate (S1P) concentration in lipoprotein fractions collected from Npc1l1^WT^ or Npc1l1^KO^ male mice (initially 8–10 weeks old) fed a Western diet (WD) for the indicated periods in the absence (−) or presence (+) of ezetimibe (Eze). Representative lipoprotein profiles from two independent experiments with two mice in each group are shown.

**Figure 3:**
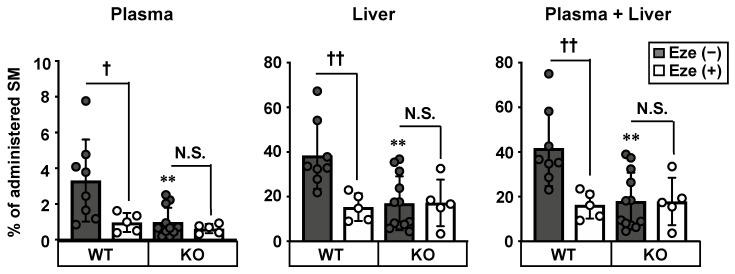
Involvement of NPC1L1 in intestinal sphingomyelin absorption. Sphingomyelin (SM) absorption was examined in 10–12 weeks-old male Npc1l1^WT^ (WT) and Npc1l1^KO^ (KO) mice treated with (+) or without (−) 0.45 mg/kg ezetimibe (Eze). [^3^H] SM concentrations in the plasma, liver, and plasma plus liver [sum of the data from the first (plasma) and second (liver) graphs] were examined 2 h after administration of [^3^H] SM-containing emulsion. Bar graphs represent the mean ± S.D. Each dot on the bar graphs represents data from a single mouse (n = 5–11). ** *p* < 0.01, significant difference by Dunnett’s test between WT mice and KO mice without ezetimibe treatment. † *p* < 0.05, †† *p* < 0.01, significant differences by Dunnett’s test comparing two groups [Eze (−) and Eze (+)]. N.S., no significant difference between groups.

Npc1l1^WT^ and Npc1l1^KO^ mice with an Ldl receptor (Ldlr) mutant background were generated as previously reported [10]. All mice were housed in temperature and humidity-controlled cages, with a 12 h light/dark cycle and with free access to water, as well as a control diet (FR-1; Funabashi Farm Co., Ltd., Chiba, Japan) or an WD (D12079B (41 kcal% fat and 0.21% cholesterol); Research Diets Inc., New Brunswick, NJ, USA). To analyze NPC1L1-dependent VLDL/LDL after WD feeding (Figure 1 and Figure 2), we administered ezetimibe (20 μg/g diets) orally by mixing with WD. For in vivo acute absorption studies (Figure 3), a single intravenous dose of ezetimibe (0.45 mg/kg body weight) was administered to mice fed a control diet. All experiments involving mice were conducted in accordance with the US National Institutes of Health Guide for the Care and Use of Laboratory Animals and with protocols approved by the Animal Studies Committee of the University of Tokyo. All the mice in this study were sacrificed by cervical dislocation.

### 2.2. Lipoprotein Fractionation

Lipoprotein fractionation was performed as described previously [10,11]. Briefly, plasma specimens collected from the mice (initially, 8–10 weeks old) after WD feeding with or without ezetimibe (20 μg/g diet) for 3 or 20 weeks were fractionated by size-exclusion chromatography using a fast protein liquid chromatography system equipped with a Superose 6 column (GE Healthcare UK Ltd., Buckinghamshire, UK). Elution was performed in PBS containing 1 mM EDTA and 3 mM sodium azide as the running buffer. After loading 300 μL of serum, the system was run at a constant flow of 200 μL/min, with 500-μL fractions collected. The cholesterol and triglyceride concentrations in each fraction were quantified with the Total Cholesterol E-test Wako and Triglyceride E-test Wako (Wako Pure Chemical Industries, Ltd., Osaka, Japan), respectively.

### 2.3. Proteomics Analysis

Plasma samples were collected from the mice (initially, 8–10 weeks old) after WD feeding with or without ezetimibe (20 μg/g diet) for 3 or 20 weeks. Collected plasma specimens were fractionated to obtain the VLDL/LDL fractions. Extraction and digestion of proteins in the VLDL/LDL fractions were performed as reported previously [12], with some modifications. Briefly, the VLDL/LDL fractions solubilized in 50 mM ammonium bicarbonate solution containing 5% sodium deoxycholate were incubated for 5 min at 95 °C and sonicated for 10 min. Next, 10 μg proteins were reduced with 5 mM Tris (2-carboxyethyl) phosphine for 20 min at room temperature, and then alkylated in the dark with 10 mM iodoacetamide for 15 min at room temperature. After adjusting the final concentrations of sodium deoxycholate to 0.5%, 0.2 μg trypsin was added to the reduced and alkylated proteins (10 μg) and the samples were incubated overnight at 37 °C. The sample solution was acidified with 0.5% trifluoroacetic acid (*v*/*v*) up to approximately pH 2.0 and centrifuged at 15,700× *g* for 10 min. The obtained supernatant containing digested peptides was concentrated and desalted with a Stage tip (Thermo Fisher Scientific, Waltham, MA, USA), according to the manufacturer’s instructions for subsequent shotgun-proteomics analyses.

Shotgun-proteomics analyses were conducted using an EASY-nLC^TM^ 1200 System connected to a Q Exactive^TM^ HF Hybrid Quadrupole-Orbitrap^TM^ Mass Spectrometer (Thermo Fisher Scientific), as reported previously [13]. Briefly, digested peptides were separated using an Acclaim^TM^ PepMap^TM^ 100 C18 LC column (75 μm × 150 mm) (Thermo Fisher Scientific) at 45 °C in a linear gradient, ranging between 0 and 35% acetonitrile containing 0.1% formic acid for 220 min at 300 nL/min, followed by a linear increase to 80% acetonitrile for 10 min, which was maintained for 25 min. Mass spectrometry was performed in data-dependent MS/MS mode with survey scanning at a resolution of 70,000 between 350 and 1500 *m*/*z*. The top 15 most abundant isotope patterns, with charge of 2–4, were selected with an isolation window of 2.0 *m*/*z*, fragmented by higher energy collisional dissociation with a normalized collision energy of 27, and analyzed at a resolution of 17,500. The maximum ion injection time for the survey scan and MS/MS scans was 60 ms; the ion target values were 3E6 and 5E5, respectively. The raw files were processed using Proteome Discoverer version 1.3 (Thermo Fisher Scientific). The fragmentation spectra were searched against Uniprot database using the MASCOT search engine. Carbamidomethylation of cysteine was set as a fixed modification; oxidation of methionine and phosphorylation of tyrosine, serine, and threonine were chosen as variable modifications for database searching. Both peptide and protein identifications were filtered at a 1% false discovery rate. Protein scores were calculated as [−10 log (P)], where P was the probability that the observed match was a random event, by using Proteome Discoverer version 1.3.

### 2.4. Quantification of S1P

Fractionated plasma samples were mixed with an internal standard solution (100 ng/mL of pioglitazone), followed by deproteinization with 4 volumes of methanol and 4 volumes of acetonitrile. After centrifugation at 15,000 rpm for 5 min, deproteinized supernatants were evaporated using a Speed-Vac concentrator (Kubota, Tokyo, Japan), and the dried samples were dissolved in methanol. To quantify the S1P concentrations in each sample, LC/MS/MS multiple reaction monitoring analyses were conducted on a XEVO^TM^ Tandem Quadrupole Mass Spectrometer coupled to an ACQUITY Ultra Performance LC (UPLC) System with an integral autoinjector (Waters, Milford, MA, USA). The XEVO spectrometer was run in electrospray ionization-MS/MS multiple reaction monitoring mode at a source temperature of 120 °C and a desolvation temperature of 350 °C. The sample temperature was maintained at 4 °C and the column temperature was maintained at 50 °C. The mobile phases were 0.1% formic acid solution (solvent A) and liquid chromatography-grade acetonitrile (Sigma Aldrich, Inc., St Louis, MO, USA) (solvent B). The UPLC and mass spectrometer conditions are shown in Supplementary Table S1.

Data analyses were performed using MassLynxNT software version 4.1 (Waters).

### 2.5. Emulsion Preparation

Emulsions were prepared as described previously [9], with minor modifications. Briefly, stock lipid solutions were mixed to give a final concentration of 13.3 mM triolein, 2.6 mM cholesterol, 3 mM Lα-phosphatidylcholine, and 3 μCi/mL [^3^H] SM: Sphingomyelin (bovine) [choline methyl-^3^H] (Muromachi Kikai, Tokyo, Japan). The solvent was evaporated, and 19 mM sodium taurocholate (dissolved in PBS) was added to obtain the required lipid concentrations. The mixture was sonicated three times for 3 min using an ultrasonic homogenizer.

### 2.6. In Vivo Acute Absorption Study

In vivo acute absorption studies were performed as described previously [9], with minor modifications. The 10–12 weeks-old male mice fed a control diet were fasted for 18 h, anesthetized with urethane, and administered an intravenous dose of 1.5 mL/kg blank plasma (for mock) or ezetimibe-containing plasma (0.3 mg/mL) via the jugular vein. Immediately after drug administration, a [^3^H] SM-containing emulsion (5 mL/kg body weight) was delivered into the small intestine via the duodenal cannula. Two hours after emulsion loading, the mice were sacrificed, and the plasma and liver were isolated to quantify the level of absorbed [^3^H] SM. The radioactivity of each specimen was measured using a liquid scintillation counter.

### 2.7. Cells

NPC1L1 overexpressing Caco-2 cells and control Caco-2 cells were generated in our previous studies by stable transfection of a pcDNA3.1(+) vector carrying human NPC1L1 cDNA with hemagglutinin (HA) tag (YPYDVPDYA) sequences at the 3′-end and an empty vector [pcDNA3.1(+)], respectively [5,7,14]. All cells were cultured in Eagle’s minimum essential medium (Nacalai Tesque, Kyoto, Japan) with 10% fetal bovine serum (Biological Industries, Beit-HaEmek, Israel), penicillin and streptomycin (100 U/mL) (Nacalai Tesque), 1% nonessential amino acids (Gibco, Grand Island, NY, USA), and G418 sulfate (500 μg/mL) (Nacalai Tesque) at 37 °C and 5% CO_2_.

### 2.8. Western Blot Analysis

Caco-2 cells were lysed with radioimmunoprecipitation assay buffer (0.1% SDS, 0.5% deoxycholate, and 1% NP-40). Total extracted cellular proteins (40 μg) were diluted with 2× SDS loading buffer and subjected to Western blot analysis, as described previously [9]. An SDS–polyacrylamide gel (7%) was used to separate the proteins in each sample. The molecular weights were determined using a prestained protein marker (New England Biolabs., Ipswich, MA, USA). The primary antibodies were 800-fold diluted rabbit anti-HA antibody [Y-11 (sc-805)] (Santa Cruz Biotechnology, Dallas, TX, USA) for human NPC1L1-HA and 1000-fold diluted rabbit anti–α-tubulin antibody (ab15246) (Cayman Chemical, Ann Arbor, MI, USA) for endogenous α-tubulin. For detection, the membrane was allowed to bind to 5000-fold diluted horseradish peroxidase–labeled anti-rabbit immunoglobulin G (IgG) antibody (NA934V) (GE Healthcare UK Ltd.) in TBS-T (Tris-buffered saline containing Tween) containing 0.1% BSA for 1 h at room temperature. Enzyme activity was assessed using an ECL Prime Western Blotting Detection Reagent (GE Healthcare UK Ltd.), with a luminescent image analyzer (Bio-Rad Laboratories, Hercules, CA, USA).

### 2.9. Preparation of SM-Containing Medium for In Vitro Uptake Assay

Cholesterol diluted in ethanol (1 μM), phosphatidylcholine diluted in methanol (50 μM), sodium taurocholate diluted in 96% ethanol (2 mM), and [^3^H] SM diluted in ethanol (0.04 μCi/mL) were mixed with or without (for mock control) ezetimibe diluted in methanol (40 μM) and then evaporated to dryness with mild heating under N_2_ gas. Transport buffer (118 mM NaCl, 23.8 mM NaHCO_3_, 4.83 mM KCl, 0.96 mM KH_2_PO_4_, 1.2 mM MgSO_4_, 12.5 mM HEPES, 5 mM glucose, and 1.53 mM CaCl_2_ adjusted to pH 7.4) was added to prepare the medium for uptake experiments. The [^3^H] SM-containing medium was thoroughly vortexed and stirred at 37 °C for 2–3 h.

### 2.10. In Vitro Uptake Assay

In vitro uptake assays were performed as described previously [5,7,9,14,15], with minor modifications. NPC1L1 overexpressing Caco-2 cells and control Caco-2 cells were seeded into 12-well plates at a density of 1.2 × 10^5^ cells/well and cultured for 14 d to allow for differentiation. During this period, the medium was replaced every 2 or 3 d. After 14 d, cells were washed twice with transport buffer and preincubated with the same buffer for 30 min. After preincubation, [^3^H] SM-containing medium was added, and the cells were incubated for 3 h. The cells were then washed with ice-cold transport buffer and disrupted with 0.2 N NaOH overnight. [^3^H] SM in the cell lysate was measured with a liquid scintillation counter to determine its cellular uptake. For normalization, the protein concentration of each well was determined using a BCA protein assay kit (Thermo Fisher Scientific).

### 2.11. Ethic Committee Approval

The project identification codes are P14-111 (approved date: 16 October 2014) and P19-052 (approved date: 6 September 2019).

## 3. Results

### 3.1. Characterization of NPC1L1-Dependent VLDL/LDL in Mice Fed WD

We analyzed lipoprotein profiles in atherosclerotic mouse models with an Ldlr mutant background after feeding with WD over the short-term (3 weeks: few atherosclerotic plaques in Npc1l1^WT^ mice were observed) and long-term (20 weeks: atherosclerotic plaques in Npc1l1^WT^ mice were cleary observed) [10]. Consistent with our previous studies [10], VLDL/LDL-cholesterol (Figure 1A), and triglyceride (Figure 1B) after long-term WD feeding were higher in Npc1l1^WT^ mice than in both Npc1l1^KO^ and ezetimibe-administered Npc1l1^WT^ mice, whereas only a minimal difference in high-density lipoprotein (HDL) lipids was observed. Notably, such an NPC1L1-dependent increase in VLDL/LDL lipids was observed even after short-term WD feeding. These results indicate that NPC1L1 controls VLDL/LDL lipids in WD-fed mice.

To further characterize the NPC1L1-dependent VLDL/LDL particles, we performed shotgun-proteomic analyses and found that the protein score of apolipoprotein M (apoM) in VLDL/LDL fractions from Npc1l1^WT^ mice was significantly higher than those from Npc1l1^KO^ and ezetimibe-administered Npc1l1^WT^ mice after both short and long-term WD feeding (Figure 1C). These results suggest that regardless of the atherosclerosis progression status, VLDL/LDL-associated apoM was increased by WD feeding in an NPC1L1-dependent manner.

### 3.2. S1P Levels in NPC1L1-Dependent VLDL/LDL in Mice Fed a WD

As apoM is a carrier of S1P on lipoproteins [16], we analyzed the lipoprotein distribution of S1P in mice after WD feeding (Figure 2). Consistent with previous studies, which showed that most of apoM is distributed to HDL in mice fed a normal diet [16], a large part of S1P was observed in the HDL fractions, whereas low levels of VLDL/LDL-associated S1P were detected in all mice tested before WD feeding. However, after WD feeding (both for 3 and 20 weeks), Npc1l1^WT^ mice exhibited increased levels of VLDL/LDL-associated S1P but decreased HDL-associated S1P. After WD feeding, Npc1l1^KO^ and ezetimibe-treated Npc1l1^WT^ mice showed little or no apparent increase in VLDL/LDL-associated S1P. These results suggest that NPC1L1 controls the diet-induced production of VLDL/LDL-associated apoM-bound S1P (VLDL/LDL-apoM-S1P).

### 3.3. Involvement of NPC1L1 in Intestinal Absorption of SM

Considering that S1P is a metabolite of SM, which is a major sphingolipid in WD, we hypothesized that NPC1L1 is involved in the intestinal absorption of dietary SM. To examine this possibility, we analyzed the intestinal absorption of SM using radioisotope ([^3^H])-labeled SM. The amount of SM absorbed in the plasma and liver was 70% (plasma), 56% (liver), and 57% (plasma + liver) lower in Npc1l1^KO^ mice than in Npc1l1^WT^ mice (Figure 3). Moreover, in Npc1l1^WT^ mice, ezetimibe reduced the absorption of SM similarly to that observed in Npc1l1^KO^ mice, whereas ezetimibe did not affect SM absorption in Npc1l1^KO^ mice.

**Figure 4:**
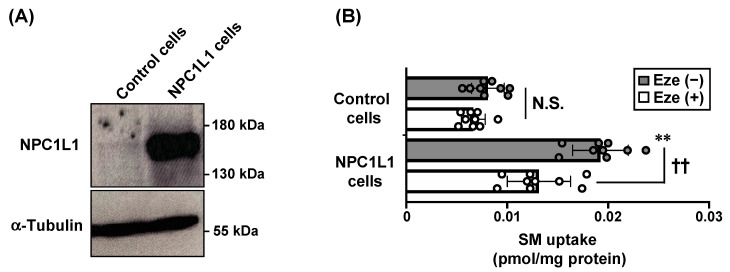
NPC1L1-mediated sphingomyelin uptake in vitro. (**A**) Protein expressions of NPC1L1-HA and α-tubulin (as a loading control) were examined in NPC1L1-overexpressing Caco-2 cells (NPC1L1 cells) and control Caco-2 cells (control cells) by Western blot analysis. (**B**) Uptake of sphingomyelin (SM) by NPC1L1 cells and control cells was examined after incubation for 3 h with [^3^H] SM-containing medium in the absence (−) or presence (+) of 40 μM ezetimibe (Eze). Bar graphs represent the mean ± S.D. Each dot on the bar graphs represents a single well of a cell culture plate from three independent experiments (n = 9). ** *p* < 0.01, significant difference by Dunnett’s test between control cells and NPC1L1 cells in the absence of ezetimibe. †† *p* < 0.01, significant difference by Dunnett’s test comparing two groups [Eze (−) and Eze (+)]. N.S., no significant difference between groups.

Consistent with in vivo observations, in vitro uptake assays using NPC1L1-overexpressing human colorectal epithelial Caco-2 cells (Figure 4A) demonstrated that the cellular uptake of SM was significantly higher in NPC1L1-overexpressing Caco-2 cells than in control Caco-2 cells, and this increase was inhibited by 40 μM ezetimibe (Figure 4B). We confirmed the low cell toxicities of the 40 μM ezetimibe in both cell lines as determined by the lack of apparent changes in cell morphologies. These results indicate that SM is an NPC1L1 substrate, and that the intestinal absorption of dietary SM is controlled by NPC1L1 in an ezetimibe-sensitive manner.

**Figure 5:**
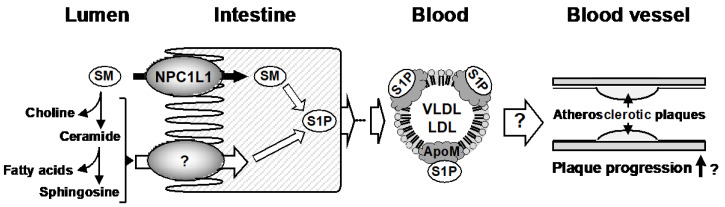
Schematic illustration of NPC1L1-mediated intestinal absorption of sphingomyelin (SM) and regulation of VLDL/LDL-apoM-sphingosine-1-phosphate (S1P). NPC1L1 would be involved in the intestinal absorption of dietary SM. It is still unclear whether SM hydrolysates such as choline, ceramide, fatty acids, and sphingosine, which are enzymatically produced from SM in the intestinal lumen, are physiological substrates of NPC1L1. After absorption, some SM is metabolized to S1P and, after several steps, S1P is exported into the blood as the apoM-bound form in VLDL/LDL. Although the (patho)physiological functions of VLDL/LDL-apoM-S1P are unclear, considering that WD-induced atherosclerotic plaques are diminished in apoM knockout mice compared to apoM wild-type mice [17], VLDL/LDL-apoM-S1P may exacerbate atherosclerosis.

Taken together, our in vivo and in vitro results demonstrate that NPC1L1 functions as an SM importer in the intestine and regulates VLDL/LDL-apoM-S1P levels in the blood (Figure 5).

## 4. Discussion

In this study, we first demonstrated that NPC1L1 regulates S1P distribution to VLDL/LDL particles in mice fed a WD. S1P is thought to be bound with apoM or albumin in the blood, and most apoM-bound S1P is distributed to HDL particles in healthy mice [16]. However, our studies with WD-induced dyslipidemia mice (Figure 1) demonstrated that a considerable amount of apoM-bound S1P was distributed to VLDL/LDL and HDL particles (Figure 2). Generally, humans have a higher serum concentration of LDL than mice because cholesteryl ester transfer protein (CETP), which is involved in transferring cholesteryl ester from HDL to LDL in the blood, is functional in humans but not in mice. Interestingly, Kurano et al., showed that the lipoprotein distribution of S1P was shifted from HDL to VLDL/LDL in mice overexpressing CETP [18]. These observations, together with our findings showing that NPC1L1 dysfunctional mice (ezetimibe-administered Npc1l1^WT^ mice and Npc1l1^KO^ mice) have lower diet-induced VLDL/LDL-apoM-S1P, indicate that CETP and NPC1L1 play key roles in regulating VLDL/LDL-apoM-S1P in humans, particularly in patients with dyslipidemia.

The physiological functions of S1P vary depending on its mode of existence in the blood. Indeed, unlike albumin-bound S1P, apoM-bound S1P on HDL (HDL-apoM-S1P) has been reported to transduce anti-inflammatory signals by activating the S1P receptor 1 in vascular endothelial cells, resulting in downregulation of proinflammatory adhesion proteins such as intracellular adhesion molecule-1 and vascular cell adhesion molecule-1 [19]. In addition to the well-known function of HDL of taking up cholesterol from lipid-laden macrophages [20], the anti-inflammatory functions of HDL-apoM-S1P contribute to the prevention of atherosclerotic disease progression. However, in contrast to the beneficial effects of HDL-apoM-S1P, Christoffersen et al., demonstrated that diet-induced atherosclerotic plaque formation was not exacerbated, but rather diminished, in apoM knockout mice [17]. This suggests that apoM has not only positive but also negative effects on atherosclerosis progression. Considering our observation that VLDL/LDL-apoM-S1P was increased in mice with dyslipidemia, it is possible that unlike HDL-apoM-S1P, VLDL/LDL-apoM-S1P has pro-atherosclerotic functions (Figure 5). Thus, studies are needed to clarify physiological functions of VLDL/LDL-apoM-S1P and important for determining the molecular mechanisms of atherosclerotic disease progression.

Another important finding of the present study is that NPC1L1 takes up SM, and intestinal absorption of dietary SM was found to be regulated by NPC1L1 in an ezetimibe-sensitive manner (Figure 3 and Figure 4). Our results, together with the previous observation that a lack of NPC1L1 affects the cellular distribution of lactosylceramide (one of glycosphingolipids), indicate that NPC1L1 is involved in sphingolipids metabolism in various manners [21]. Although we demonstrate the potential involvement of NPC1L1 in SM absorption, there are some limitations in our study. First, because our in vivo acute absorption study (Figure 3) used [^3^H]-choline-labeled SM, we were unable to discriminate the absorption of intact SM from that of SM-derived choline, which is enzymatically produced from SM in the intestinal lumen; current understanding of SM digestion and absorption is that luminal sphingomyelin is hydrolyzed in the middle-to-distal small intestine by alkaline sphingomyelinase in the intestinal lumen, to yield ceramide and phosphocholine [22]. Phosphocholine is hydrolyzed by phosphatases in the gastrointestinal tract and is absorbed as choline, whereas ceramide is hydrolyzed by brush border neutral ceramidase to produce sphingosine and a fatty acid (Figure 5). Given these facts—together with previous observation that intact SM is not thought to be absorbed efficiently, but its sequentially hydrolyzed products (i.e., choline, fatty acid, sphingosine) are more likely to be absorbed [23]—it is possible that our in vivo SM absorption study (Figure 3) observed not only intact SM absorption, but also SM-derived choline absorption. Further studies are warranted to determine whether choline is a substrate of NPC1L1. In addition, experiments using radiolabeled sphingosine moiety of SM would be helpful to further clarify the involvement of NPC1L1 in the intestinal absorption of SM and SM-derived sphingolipids, such as ceramide and sphingosine.

Second, detailed molecular mechanisms of NPC1L1-mediated SM uptake remain unknown. Regarding the NPC1L1-mediated uptake of cholesterol, it has been reported that N-terminal domain of NPC1L1 binds cholesterol and promotes the formation of cholesterol-enriched microdomain in the plasma membrane to facilitate cholesterol transport [24,25]. In addition, recent cryo-electron microscopy structural analyses indicated that NPC1L1 forms a cholesterol-delivering tunnel from the N-terminal domain to the plasma membrane and that ezetimibe binds to the middle of the tunnel, resulting in the inhibition of cholesterol uptake [26]. As sphingolipids are colocalized with cholesterol in the plasma membrane [27], it is possible that NPC1L1 promotes the incorporation of sphingolipids, as well as cholesterol, into the plasma membrane to facilitate their transport. Previous findings, showing that excess sphingolipids and cholesterol mutually inhibit each other’s absorption [28,29], support our hypothesis that sphingolipids and cholesterol share the same mechanism for their intestinal absorption. Another possibility is that NPC1L1 might indirectly affect SM absorption, rather than being directly responsible for SM transport. The lower absorption of SM in Npc1l1^KO^ mice and ezetimibe-administered Npc1l1^WT^ mice (Figure 3) is consistent with the lower absorption of cholesterol [4,9], so it is possible that the decreased SM absorption in NPC1L1 dysfunctional mice is due to the indirect effect via increased luminal cholesterol in the absence of functional NPC1L1, resulting in increased cholesterol-SM interaction to inhibit each other’s absorption [27,28,29]. Further studies to clarify the direct binding capacity and binding site(s) of NPC1L1 to SM would be valuable for revealing the underlying mechanisms.

## 5. Conclusions

In conclusion, our results indicate that NPC1L1, a cholesterol importer, is involved in the uptake of dietary SM and controls diet-induced VLDL/LDL-apoM-S1P levels in the blood (Figure 5). This is the first study to identify the physiological transporter of sphingolipids, except for S1P exporters [30,31]. To understand the physiological functions of NPC1L1 and pharmacological effects of ezetimibe, comprehensive analyses of plasma lipids, particularly VLDL/LDL components, are needed.

## Supplementary Materials

The following are available online at https://www.mdpi.com/2072-6643/12/9/2641/s1, Table S1. UPLC-MS/MS conditions to determine S1P amounts.

## Abbreviations

apoM	apolipoprotein M
HDL	high-density lipoprotein
LDL	low-density lipoprotein
NPC1L1	Niemann-Pick C1-Like 1
SM	Sphingomyelin
S1P	sphingosine-1-phosphate
VLDL	very low-density lipoprotein
WD	Western diet

## References

[1] Hannun Y.A., Obeid L.M. (2008). Principles of bioactive lipid signalling: Lessons from sphingolipids. Nat. Rev. Mol. Cell Biol..

[2] Norris G.H., Blesso C.N. (2017). Dietary sphingolipids: Potential for management of dyslipidemia and nonalcoholic fatty liver disease. Nutr. Rev..

[3] Dei Cas M., Ghidoni R. (2018). Cancer Prevention and Therapy with Polyphenols: Sphingolipid-Mediated Mechanisms. Nutrients.

[4] Altmann S.W., Davis H.R., Zhu L.J., Yao X., Hoos L.M., Tetzloff G., Iyer S.P., Maguire M., Golovko A., Zeng M. (2004). Niemann-Pick C1 Like 1 protein is critical for intestinal cholesterol absorption. Science.

[5] Yamanashi Y., Takada T., Suzuki H. (2007). Niemann-Pick C1-like 1 overexpression facilitates ezetimibe-sensitive cholesterol and beta-sitosterol uptake in CaCo-2 cells. J. Pharmacol. Exp. Ther..

[6] Garcia-Calvo M., Lisnock J., Bull H.G., Hawes B.E., Burnett D.A., Braun M.P., Crona J.H., Davis H.R., Dean D.C., Detmers P.A. (2005). The target of ezetimibe is Niemann-Pick C1-Like 1 (NPC1L1). Proc. Natl. Acad. Sci. USA.

[7] Narushima K., Takada T., Yamanashi Y., Suzuki H. (2008). Niemann-pick C1-like 1 mediates alpha-tocopherol transport. Mol. Pharmacol..

[8] Takada T., Suzuki H. (2010). Molecular mechanisms of membrane transport of vitamin E. Mol. Nutr. Food Res..

[9] Takada T., Yamanashi Y., Konishi K., Yamamoto T., Toyoda Y., Masuo Y., Yamamoto H., Suzuki H. (2015). NPC1L1 is a key regulator of intestinal vitamin K absorption and a modulator of warfarin therapy. Sci. Transl. Med..

[10] Yamamoto H., Yamanashi Y., Takada T., Mu S., Tanaka Y., Komine T., Suzuki H. (2019). Hepatic Expression of Niemann-Pick C1-Like 1, a Cholesterol Reabsorber from Bile, Exacerbates Western Diet-Induced Atherosclerosis in LDL Receptor Mutant Mice. Mol. Pharmacol..

[11] Yamamoto H., Takada T., Yamanashi Y., Ogura M., Masuo Y., Harada-Shiba M., Suzuki H. (2017). VLDL/LDL acts as a drug carrier and regulates the transport and metabolism of drugs in the body. Sci. Rep..

[12] Lin Y., Liu Y., Li J., Zhao Y., He Q., Han W., Chen P., Wang X., Liang S. (2010). Evaluation and optimization of removal of an acid-insoluble surfactant for shotgun analysis of membrane proteome. Electrophoresis.

[13] Ikebuchi Y., Aoki S., Honma M., Hayashi M., Sugamori Y., Khan M., Kariya Y., Kato G., Tabata Y., Penninger J.M. (2018). Coupling of bone resorption and formation by RANKL reverse signalling. Nature.

[14] Yamanashi Y., Takada T., Suzuki H. (2009). In-vitro characterization of the six clustered variants of NPC1L1 observed in cholesterol low absorbers. Pharm. Genom..

[15] Ito S.M., Yamanashi Y., Takada T., Suzuki H. (2019). Clinical Importance of Drug-Drug Interaction Between Warfarin and Prednisolone and Its Potential Mechanism in Relation to the Niemann-Pick C1-Like 1-Mediated Pathway. Circ. J..

[16] Christoffersen C., Obinata H., Kumaraswamy S.B., Galvani S., Ahnstrom J., Sevvana M., Egerer-Sieber C., Muller Y.A., Hla T., Nielsen L.B. (2011). Endothelium-protective sphingosine-1-phosphate provided by HDL-associated apolipoprotein M. Proc. Natl. Acad. Sci. USA.

[17] Christoffersen C., Pedersen T.X., Gordts P.L., Roebroek A.J., Dahlback B., Nielsen L.B. (2010). Opposing effects of apolipoprotein m on catabolism of apolipoprotein B-containing lipoproteins and atherosclerosis. Circ. Res..

[18] Kurano M., Hara M., Ikeda H., Tsukamoto K., Yatomi Y. (2017). Involvement of CETP (Cholesteryl Ester Transfer Protein) in the Shift of Sphingosine-1-Phosphate Among Lipoproteins and in the Modulation of its Functions. Arterioscler. Thromb. Vasc. Biol..

[19] Galvani S., Sanson M., Blaho V.A., Swendeman S.L., Obinata H., Conger H., Dahlback B., Kono M., Proia R.L., Smith J.D. (2015). HDL-bound sphingosine 1-phosphate acts as a biased agonist for the endothelial cell receptor S1P1 to limit vascular inflammation. Sci. Signal..

[20] Ouimet M., Barrett T.J., Fisher E.A. (2019). HDL and Reverse Cholesterol Transport. Circ. Res..

[21] Davies J.P., Scott C., Oishi K., Liapis A., Ioannou Y.A. (2005). Inactivation of NPC1L1 causes multiple lipid transport defects and protects against diet-induced hypercholesterolemia. J. Biol. Chem..

[22] Norris G.H., Milard M., Michalski M.C., Blesso C.N. (2019). Protective properties of milk sphingomyelin against dysfunctional lipid metabolism, gut dysbiosis, and inflammation. J. Nutr. Biochem..

[23] Nilsson A., Duan R.D. (2006). Absorption and lipoprotein transport of sphingomyelin. J. Lipid. Res..

[24] Zhang J.H., Ge L., Qi W., Zhang L., Miao H.H., Li B.L., Yang M., Song B.L. (2011). The N-terminal domain of NPC1L1 protein binds cholesterol and plays essential roles in cholesterol uptake. J. Biol. Chem..

[25] Li P.S., Fu Z.Y., Zhang Y.Y., Zhang J.H., Xu C.Q., Ma Y.T., Li B.L., Song B.L. (2014). The clathrin adaptor Numb regulates intestinal cholesterol absorption through dynamic interaction with NPC1L1. Nat. Med..

[26] Huang C.S., Yu X., Fordstrom P., Choi K., Chung B.C., Roh S.H., Chiu W., Zhou M., Min X., Wang Z. (2020). Cryo-EM structures of NPC1L1 reveal mechanisms of cholesterol transport and ezetimibe inhibition. Sci. Adv..

[27] Slotte J.P. (1999). Sphingomyelin-cholesterol interactions in biological and model membranes. Chem. Phys. Lipids.

[28] Noh S.K., Koo S.I. (2003). Egg sphingomyelin lowers the lymphatic absorption of cholesterol and alpha-tocopherol in rats. J. Nutr..

[29] Nyberg L., Duan R.D., Nilsson A. (2000). A mutual inhibitory effect on absorption of sphingomyelin and cholesterol. J. Nutr. Biochem..

[30] Kawahara A., Nishi T., Hisano Y., Fukui H., Yamaguchi A., Mochizuki N. (2009). The sphingolipid transporter spns2 functions in migration of zebrafish myocardial precursors. Science.

[31] Vu T.M., Ishizu A.N., Foo J.C., Toh X.R., Zhang F., Whee D.M., Torta F., Cazenave-Gassiot A., Matsumura T., Kim S. (2017). Mfsd2b is essential for the sphingosine-1-phosphate export in erythrocytes and platelets. Nature.

