# Supplementary material for: NPC1L1 Facilitates Sphingomyelin Absorption and Regulates Diet-Induced Production of VLDL/LDL-associated S1P
Source: Nutrients. 2020 Aug 30;12(9):2641. doi: 10.3390/nu12092641 (PMC7551898; doi:10.3390/nu12092641)
Supplement: Supplementary file 1 [file nutrients-12-02641-s001.pdf]

Table S1. UPLC-MS/MS conditions. The UPLC-MS/MS conditions to determine sphingosine-1-phosphate (S1P) amounts.

Compounds	Retention Time	Detection Conditions	Elution Condition
**S1P**	4.14 [min]	Parent Mass : 380.38<br>Daughter Mass : 264.22<br>Cone Voltage : 20V<br>Collision Energy : 15V	**Solvent A** : water with 0.1% formic acid<br>**Solvent B** : Acetonitrile with 0.1% formic acid<br>**Flow Rate** : 0.3 [mL/min]
**Pioglitazone (internal standard)**	3.01 [min]	Parent Mass : 357.15<br>Daughter Mass : 134.0<br>Cone Voltage : 28V<br>Collision Energy : 16V	**Elution Method**<br>Time [min] / Solvent A [%] / Solvent B [%]<br>0 / 100 / 0<br>1 / 100 / 0<br>3.5 / 2 / 98<br>5.0 / 2 / 98<br>5.1 / 100 / 0
